# 非小细胞肺癌细胞系PC-9耐药突变细胞株的建立及其对EGFR抑制剂的敏感性验证

**DOI:** 10.3779/j.issn.1009-3419.2024.101.31

**Published:** 2024-11-20

**Authors:** Tao HU, Yang LOU, Mingbo SU

**Affiliations:** ^1^230012 合肥，安徽中医药大学药学院（胡涛）; ^1^School of Pharmacy, Anhui University of Chinese Medicine, Hefei 230012, China; ^2^226133 南通，长三角药物高等研究院（胡涛，苏明波）; ^2^Yangtze Delta Drug Advanced Research Institute, Nantong 226133, China; ^3^226133 南通，南通弘烨医药科技有限公司（胡涛，楼洋，苏明波）; ^3^Nantong Hongye Pharmaceutical Technology Co., Ltd, Nantong 226133, China

**Keywords:** 肺肿瘤, CRISPR/Cas9技术, EGFR突变, EGFR抑制剂, Lung neoplasms, CRISPR/Cas9 technology, EGFR mutation, EGFR inhibitors

## Abstract

**背景与目的:**

表皮生长因子受体（epidermal growth factor receptor, EGFR）激酶结构域的突变是非小细胞肺癌（non-small cell lung cancer, NSCLC）的关键发病因素，小分子EGFR-酪氨酸激酶抑制剂（tyrosine kinase inhibitors, TKIs）是治疗EGFR突变NSCLC的一线药物，而EGFR的耐药突变限制了EGFR-TKIs的临床应用。本研究旨在利用簇状规则间隔短回文重复序列（clustered regularly interspaced short palindromic repeats, CRISPR）/CRISPR相关核酸酶9（CRISPR-associated nuclease 9, Cas9）基因敲入技术，构建贴近临床的具有EGFR^D19/T790M/C797S^突变类型的PC-9 EGFR^D19/T790M/C797S^细胞模型，用于研究小分子EGFR-TKIs的抑制作用及为新一代靶向EGFR突变耐药的创新药物研发提供细胞载体。

**方法:**

利用CRISPR/Cas9技术在EGFR^D19^突变类型的人NSCLC细胞PC-9上敲入EGFR^T790M/C797S^突变片段，构建具有EGFR^D19/T790M/C797S^突变类型的PC-9 EGFR^D19/T790M/C797S^细胞模型，以MTS法验证EGFR-TKIs对其增殖抑制作用，以Western blot证明EGFR蛋白表达以及下游蛋白激酶B（protein kinase B, AKT）和丝裂原活化蛋白激酶（mitogen-activated protein kinase, MAPK）磷酸化调控。

**结果:**

成功构建EGFR^D19/T790M/C797S^突变的PC-9 EGFR^D19/T790M/C797S^细胞株，在增殖抑制上，已上市的第一、第二及第三代对EGFR^D19/T790M/C797S^突变无效的EGFR-TKIs对该细胞株的增殖抑制活性均较弱，增殖抑制半抑制浓度（half maximal inhibitory concentration, IC_50_）值均大于1000 nmol/L，而对EGFR^D19/T790M/C797S^突变具有较好疗效的在研的第四代EGFR-TKIs对该细胞模型具有较强的增殖抑制作用；在机制验证上，第一代、第二和第三代EGFR-TKIs对该细胞株EGFR及下游AKT/MAPK信号通路的磷酸化抑制活性均弱，而在研第四代EGFR-TKIs可显著抑制该细胞株EGFR及下游AKT/MAPK信号通路的磷酸化。

**结论:**

利用CRISPR/Cas9技术在PC-9细胞上敲入EGFR^T790M/C797S^突变片段成功构建含EGFR^D19/T790M/C797S^突变的细胞株，并验证其对EGFR^D19/T790M/C797S^突变是否有效的EGFR-TKIs表现出不同的敏感性且对EGFR及下游通路磷酸化表现出不同的抑制作用，证明该细胞株依赖EGFR^D19/T790M/C797S^突变及EGFR/AKT/MAPK信号通路的激活进行增殖。本研究为新一代靶向EGFR突变耐药的创新药物研发提供了贴近临床的细胞水平的活性评价和机制验证体系。

## Abstract (en)

**Background and objective:**

Mutations in the structural domain of the epidermal growth factor receptor (EGFR) kinase represent a critical pathogenetic factor in non-small cell lung cancer (NSCLC). Small-molecule EGFR-tyrosine kinase inhibitors (TKIs) serve as first-line therapeutic agents for the treatment of EGFR-mutated NSCLC. But the resistance mutations of EGFR restrict the clinical application of EGFR-TKIs. In this study, we constructed a clinically relevant PC-9 EGFR^D19/T790M/C797S^ cellular model featuring the mutation type within the EGFR^D19/T790M/C797S^. This model aims to investigate the inhibitory effects of small-molecule EGFR-TKIs and to provide a cellular platform for developing a new generation of innovative drugs that target resistance associated with EGFR mutations.

**Methods:**

Clustered regularly interspaced short palindromic repeats/CRISPR-associated nuclease 9 (CRISPR/Cas9) technology was employed to knock in the EGFR^T790M/C797S ^mutant fragment into NSCLC PC-9 cells, originally harboring the EGFR^D19^ mutation, to generate the PC-9 EGFR^D19/T790M/C797S^ cell model. This model, with the EGFR^D19/T790M/C797S^ mutant, was used to investigate the inhibitory effects of EGFR-TKIs on cell proliferation through MTS assay. Additionally, Western blot analysis was conducted to assess the regulation of EGFR protein expression and the phosphorylation levels of downstream signaling molecules, including protein kinase B (AKT) and mitogen-activated protein kinase (MAPK).

**Results:**

PC-9 EGFR^D19/T790M/C797S^ cells, with the EGFR^D19/T790M/C797S^ mutation, were successfully generated using CRISPR/Cas9 technology. In terms of proliferation inhibition, the marketed first-, second-, and third-generation EGFR-TKIs that were ineffective against the EGFR^D19/T790M/C797S^ mutation showed weak proliferation inhibitory activity against this cell line, and the proliferation inhibition (half maximal inhibitory concentration, IC_50_)>1000 nmol/L; in contrast, the fourth-generation EGFR-TKIs in development, which have better efficacy against the EGFR^D19/T790M/C797S^ mutation, showed strong proliferation inhibition in this cell model. On mechanistic validation, the first-, second-, and third-generation EGFR-TKIs had weak inhibitory activity on the phosphorylation of EGFR and the downstream AKT/MAPK signaling pathway in this cell line, whereas the fourth generation of EGFR-TKIs under development significantly inhibited the phosphorylation of EGFR and the downstream AKT/MAPK signaling pathway in this cell line.

**Conclusion:**

Using CRISPR/Cas9 technology, the EGFR^T790M/C797S^ mutant fragment was successfully knocked into PC-9 cells to create cell lines harboring the EGFR^D19/T790M/C797S^ mutation. The study demonstrated that the EGFR-TKIs showed different sensitivities to whether the EGFR^D19/T790M/C797S^ mutation was effective or not and different inhibitory effects on the phosphorylation of EGFR and downstream pathways, which demonstrated that this cell line depended on the activation of the EGFR^D19/T790M/C797S^ mutation and EGFR/AKT/MAPK signaling pathway for proliferation. This study provides a clinically relevant cellular evaluation and mechanism validation system for the development of a new generation of innovative drugs targeting EGFR mutation resistance.

2022年，全球有将近2000万新发癌症病例和970多万癌症死亡病例，肺癌的发病率和死亡率均排名第一，预估2024年肺癌依旧是死亡率排名最高的癌症^[1]^。临床研究^[2]^表明，表皮生长因子受体（epidermal growth factor receptor, EGFR）激酶结构域的突变是非小细胞肺癌（non-small cell lung cancer, NSCLC）的关键发病因素。EGFR信号通路对细胞的生长、增殖和分化等生理过程发挥重要的作用，EGFR发生致病突变时会自磷酸化，并使下游蛋白激酶B（protein kinase B, AKT）/丝裂原活化蛋白激酶（mitogen-activated protein kinase, MAPK）信号通路持续磷酸化激活，从而导致细胞异常增殖^[3,4]^。NSCLC患者的EGFR突变类型中EGFR 19号外显子的缺失突变（D19）和21号外显子的点突变（L858R）占据了主要比例^[5,6]^。由于已有针对这两种突变的小分子酪氨酸激酶抑制剂（tyrosine kinase inhibitors, TKIs）上市，因而又称之为敏感突变。现有的EGFR-TKIs分为第一、第二和第三代抑制剂，其中第一和第二代抑制剂均针对敏感突变，并随着广泛的使用而出现了D19/T790M和L858R/T790M耐药双突变^[7,8]^。第三代抑制剂如奥希替尼（Osimertinib），能够同时针对敏感突变和T790M耐药突变，已成为EGFR突变阳性NSCLC患者的一线首选药物^[9⇓-11]^。但近年来在临床中也发现了Osimertinib耐药的患者，进一步研究^[12⇓⇓-15]^表明其主要的耐药机制是EGFR^C797S^耐药突变，目前针对EGFR^C797S^突变类型进行了第四代EGFR-TKIs的开发，已发现了一些在临床前实验中对EGFR^C797S^突变具有良好活性的EGFR-TKIs分子，如TQB3804^[16]^和BI4020^[17]^，但目前还没有针对EGFR^D19/T790M/C797S^和EGFR^L858R/T790M/C797S^的批准药物。因此，第四代EGFR-TKIs的患者群体巨大，前景广阔，构建用于第四代EGFR-TKIs的体内外药效学评价体系具有重大意义。

在EGFR-TKIs的开发过程中，Ba/F3细胞模型在阐明分子靶向药物治疗后产生的耐药机制方面发挥了重要作用，携带驱动突变和继发突变的Ba/F3细胞可用于评估药物敏感性或研究可以克服初始耐药性的药物^[18]^。然而，应该注意的是，Ba/F3模型有一些局限性：首先，通常难以控制转染驱动基因的表达水平（以及引入的基因拷贝数）；其次，由于通常只将单个驱动突变引入Ba/F3细胞，因此已建立的Ba/F3细胞系不能反映临床中EGFR突变肿瘤细胞内较为复杂的突变情况。因此，构建贴近临床的细胞工具用于新一代靶向EGFR耐药突变的创新药物的研发至关重要。

簇状规则间隔短回文重复序列（clustered regularly interspaced short palindromic repeats, CRISPR）/ CRISPR相关核酸酶9（CRISPR-associated nuclease 9, Cas9）是一种由RNA指导的Cas9核酸酶对靶向基因进行编辑的技术^[19]^，即小向导RNA（single-guide RNA, sgRNA）指导Cas9蛋白在靶定位点切断双链DNA，在细胞自身存在的DNA损伤修复机制下，序列重新连接，在修复过程出现片段插入或突变等情况，从而进行编辑^[20]^。利用该技术，在人NSCLC细胞中将基因组DNA编辑为EGFR耐药突变类型，而并非采取转入外源基因的方式构建细胞评价模型，可构建出贴近临床的细胞工具，从而避免在Ba/F3细胞中的局限性，并可丰富在新一代靶向EGFR耐药突变的创新药物研发中的细胞水平活性评价及机制验证体系。

本研究利用CRISPR/Cas9技术，构建具有EGFR^D19/T790M/C797S^突变的PC-9 EGFR^D19/T790M/C797S^细胞，利用Western blot法评价不同EGFR-TKIs对该细胞的EGFR及下游AKT/MAPK信号通路磷酸化的抑制作用，利用MTS法评价不同EGFR-TKIs对该细胞的增殖抑制作用，以期为新一代靶向EGFR突变耐药的创新药物研发提供贴近临床的细胞水平的活性评价和机制验证体系。

## 1 材料与方法

### 1.1 一般资料

人胚肾细胞HEK-293T（生产厂家：ATCC）；人NSCLC细胞PC-9（生产厂家：ATCC）；DH5α化学感受态细胞（生产厂家：诺唯赞生物科技股份有限公司）。

pCMV-T7-SpRY-HF1-P2A-EGFP（生产厂家：RTW5008）、Cas9质粒（货号：P17206）、pU6-Bsa I-sgRNA-acceptor质粒（货号：P12226）由武汉淼灵生物科技有限公司提供；快速内切酶Bsa I（生产厂家：Absin，货号：abs60206）；Hieff^®^ Quick T4 DNA Ligase（生产厂家：Yeasen，货号：10301ES42）；PEIpro®转染试剂（生产厂家：Polyplus，货号：101000029）；T7核酸内切酶I（生产厂家：Vazyme，货号：EN303-02）；jetPRIME®转染试剂（生产厂家：Polyplus，货号：101000046）；DMEM和RPMI-1640培养基（生产厂家：Corning，货号分别为10-016-CV和10-040-CV）；胎牛血清（生产厂家：Sigma，货号：F8318）；硫酸链霉素和青霉素G钠盐（生产厂家：TCI，货号分别为S0585和P1770）；NU7441（生产厂家：MCE，货号：#HY-11006）；Gefitinib（生产厂家：MCE，货号：#HY-50895）；Afatinib（生产厂家：MCE，货号：#HY-10261）；Osimertinib（生产厂家：Aladdin-e，货号：A302849）；TQB3804和BI4020由南通弘烨医药科技有限公司提供。CellTiter 96® AQueous One Solution Cell Proliferation Assay试剂盒（MTS细胞活性检测试剂）（生产厂家：上海盛兆生物科技有限公司，货号：G3581）；GAPDH抗体（生产厂家：Proteintech，货号：60004-1-Ig）；EGFR抗体（生产厂家：Santa Cruz Biotechnology，货号：J2720）；p-EGFR抗体（生产厂家：CST，货号：3777S）；AKT抗体（生产厂家：CST，货号：9272S）；p-AKT抗体（生产厂家：CST，货号：4060S）；p44/42MAPK抗体（生产厂家：CST，货号：9102S）；p-p44/42MAPK抗体（生产厂家：CST，货号：9101S）；山羊抗鼠IgG和山羊抗兔IgG（生产厂家：Jackson IR，货号分别为111-035-003和111-035-144）；ECL显影液（生产厂家：Thermo Scientific，货号：34580）。

CO_2_细胞培养箱（生产厂家：Thermo Scientific公司，型号：4111）；多功能酶标仪（生产厂家：PerkinElmer公司，型号：HH35000310）；电泳仪（生产厂家：Bio-Red公司，型号：PowerPac HC）；凝胶成像仪（生产厂家：Bio-Red公司，型号：ChemiDoc MP）；高速冷冻离心机（生产厂家：Scilogex公司，型号：D3024R）。

### 1.2 方法

#### 1.2.1 细胞培养

HEK-293T细胞（贴壁细胞）采用含10% FBS、100 U/mL青霉素和100 μg/mL链霉素的DMEM（含4.5 g/L葡萄糖，4 mmol/L谷氨酰胺，1 mmol/L丙酮酸钠）培养基进行培养，培养条件为37 °C、5% CO_2_，取对数生长期的细胞用于后续实验。PC-9细胞（贴壁细胞）采用含10% FBS、100 U/mL青霉素和100 μg/mL链霉素的RPMI-1640培养基进行培养，培养条件为37 °C、5% CO_2_，取对数生长期的细胞用于后续实验。

#### 1.2.2 药物配制

药物母液配制：称取NU7441、Gefitinib、Afatinib、Osimertinib、TQB3804和BI4020粉末，计算后加入二甲基亚砜（dimethyl sulfoxide, DMSO）配成10 mmol/L母液，分装后置于-80 ^o^C冰箱待用。

#### 1.2.3 CRISPR/Ca9介导的基因敲入方法的建立

##### 1.2.3.1 sgRNA质粒的构建与鉴定

利用sgRNA设计网站（zlab.squarespace.com）设计靶向EGFR^Cys797^对应位点附近的sgRNA序列和EGFR^T790M/C797S^突变类型的同源修复（homology directed repair, HDR）模板序列，利用脱靶效率预测分析工具（https://cm.jefferson.edu/Off-Spotter/）筛选出脱靶效率较低的sgRNA序列，sgRNA序列如表1所述，HDR模板序列为：TGATGGCCAGCGTGGACAACCCCCACGTGTGCCGCCTGCTGGGCATCTGCCTCACCTCCACCGTGCAGCTCATCATGCAGCTCATGCCCTTCGGCAGCCTGCTCGATTACGTGAGAGAGCACAAAGACAATATTGGCTCCCAGTACCTGCTCAACTGGTGTGTGCAGATCGCAAAGGTAA。

**表1:** sgRNA及基因组DNA PCR引物序列

Name		Sequence（5’-3’）
EGFR C797+1T	Forword	CACCGACATAGTCCAGGAGGCAGC
	Reverse	AAACGCTGCCTCCTGGACTATGTC
EGFR C797+4T	Forword	CACCGCCGGACATAGTCCAGGAGGC
	Reverse	AAACGCCTCCTGGACTATGTCCGGC
EGFR C797+16T	Forword	CACCGTCTTTGTGTTCCCGGACAT
	Reverse	AAACATGTCCGGGAACACAAAGAC
EGFR C797+6C	Forword	CACCGTTCCCGGACATAGTCCAGG
	Reverse	AAACCCTGGACTATGTCCGGGAAC
EGFR C797+9C	Forword	CACCGTGTTCCCGGACATAGTCC
	Reverse	AAACGGACTATGTCCGGGAACAC
EGFR exon 20	Forword	CCCATTGTGTGCCTGATTGC
	Reverse	ACTGACTGCCACTGCAACC

sgRNA: single-guide RNA; PCR: polymerase chain reaction; EGFR: epidermal growth factor receptor.

利用Bsa I限制性内切酶切割pU6-Bsa I-sgRNA-acceptor质粒构建线性化载体，将线性化载体与表中各引物用T4 DNA连接酶连接后获得sgRNA质粒，产物以琼脂糖凝胶电泳分离并以胶回收纯化，利用DH5α化学感受态细胞进行转化涂板，挑选单克隆菌株扩增后抽提纯化质粒，本研究引物合成均由上海赛恒生物科技有限公司提供。

##### 1.2.3.2 sgRNA的筛选

使用PEIpro®转染试剂，分别将6种sgRNA质粒和Cas9质粒物质的量之比为1:1的比例共转染HEK-293T细胞，48 h后收取细胞抽提基因组DNA后使用表1中EGFR外显子20引物进行聚合酶链式反应（polymerase chain reaction, PCR）扩增，产物用T7核酸内切酶I（T7 endonuclease I）切割后以琼脂糖凝胶电泳分离，验证各sgRNA的编辑效率，编辑效率最高的sgRNA为最适sgRNA。

#### 1.2.4 PC-9 EGFR^D19/T790M/C797S^突变工程细胞株的构建与鉴定

##### 1.2.4.1 PC-9上CRISPR/Cas9敲入EGFR^T790M/C797S^突变

PC-9细胞以1.8×10^5^个/孔接入12孔板，培养18 h；将sgRNA质粒、Cas9质粒以物质的量比为1:1的比例混合后，取0.8 μg与30 ng的HDR模板序列加入至100 μL jetPRIME buffer，涡旋混合5 s后，瞬时离心1 s重新收集试剂；加入2 μL jetPRIME转染试剂，移液枪吹打3下后涡旋1 s，瞬时离心1 s重新收集；室温孵育10 min后加入12孔板内，逐滴加入，轻柔地摇晃孔板使混合均匀；转染24 h后加入作用浓度为5 μmol/L NU7441处理24 h，48 h后加入500 nmol/L Osimertinib筛选出具有Osimertinib耐药性的细胞。

##### 1.2.4.2 PC-9 EGFR^D19/T790M/C797S^细胞的单克隆挑选及验证

筛选后获得的细胞挑选出16个单克隆，以MTS法验证对Osimertinib和BI4020的敏感性差异，从而挑选出最适宜用于评价EGFR-TKIs的单克隆。提取该单克隆及PC-9细胞的基因组DNA，使用表1中EGFR外显子20引物进行PCR扩增后送至苏州金唯智生物科技有限公司测序，测序结果利用Synthego Inference of CRISPR Edits（ICE）分析工具（www.synthego.com）分析HDR模板敲入效率，验证是否具有EGFR^D19/T790M/C797S^突变类型^[21]^。

#### 1.2.5 PC-9EGFR^D19/T790M/C797S^突变工程细胞株对EGFR抑制剂的响应

##### 1.2.5.1 MTS法检测EGFR抑制剂对PC-9EGFR^D19/T790M/C797S^及PC-9细胞的增殖抑制活性

将PC-9 EGFR^D19/T790M/C797S^及PC-9细胞以500个/孔接种于96孔培养板，加入终浓度分别为10,000、1000、200、40、8、1.6、0.32和0.064 nmol/L的化合物。每个浓度设3个复孔，并设相应浓度的DMSO（2‰）溶剂对照以及纯完全培养基对照。加药后在37 ^o^C、5% CO_2_条件下培养72 h后，向每孔中加入MTS试剂20 µL，37 ^o^C下孵育1-2 h，混匀后用酶标仪测定490和690 nm处的光密度（optical density, OD）值。按下列公式计算被测药物对细胞作用后的活性百分数（Activity%）。用OD_1_表示含细胞的给药孔的吸光值（OD_490_-OD_690_），OD_2_表示不含细胞的给药孔的吸光值（OD_490_-OD_690_），OD_3_表示含细胞的DMSO孔的吸光值（OD_490_-OD_690_）。化合物作用后细胞活率（Activity%）计算公式：Activity%=（OD_1_-OD_2_）/（OD_3_-OD_2_）×100。以浓度的对数值对活性百分数（Activity%）作图，采用非线性回归算出拟合曲线，利用GraphPad Prism5软件log（inhibitor）vs normalized response-Variable slope参数设置计算得到半抑制浓度（half maximal inhibitory concentration, IC_50_）值。

##### 1.2.5.2 Western blot法检测EGFR抑制剂对PC-9EGFR^D19/T790M/C797S^及PC-9细胞的EGFR及下游磷酸化抑制活性

将PC-9 EGFR^D19/T790M/C797S^及PC-9细胞以2.5×10^5^个/孔接种于12孔板中，加入终浓度分别为200与40 nmol/L的各个EGFR抑制剂，以2‰ DMSO为空白对照组，孵育6 h后吸去上清，加入Loading buffer冰上裂解30 min，收集裂解后的样品于金属浴95 ^o^C煮15 min。样品冷却后进行SDS-PAGE电泳，转至硝酸纤维膜，5%脱脂奶粉室温封闭1.5 h，向对应条带加入EGFR、p-EGFR、AKT、p-AKT、p44/42MAPK、p-p44/42MAPK和GAPDH抗体4 ^o^C孵育过夜，清洗3次后加入相应山羊抗鼠IgG和山羊抗兔IgG抗体室温孵育1 h，清洗3次后加入ECL显影液，置于凝胶成像仪进行曝光。

### 1.3 统计学方法

增殖抑制数据采用GraphPad Prism5进行统计分析，结果以均数±标准差表示。蛋白磷酸化抑制活性测试结果采用Image Lab6对各条带进行灰度扫描，结果采用GraphPad Prism5进行统计分析，组间的差异比较使用单因素方差分析（One-Way ANOVA），P<0.05表示差异有统计学意义。

## 2 结果

### 2.1 CRISPR/Ca9介导的基因敲入方法的建立

各sgRNA产物琼脂糖凝胶电泳分离结果如图1A所示，产物胶回收纯化后利用DH5α化学感受态细胞进行转化涂板，挑选单克隆菌株扩增后进行质粒抽提，获得各sgRNA质粒命名为EGFR C797+1T、EGFR C797+4T、EGFR C797+16T、EGFR C797+6C及EGFR C797+9C。将sgRNA质粒与Cas9质粒共转染HEK239T细胞，48 h后提取细胞基因组DNA并扩增外显子20区域用于编辑效率鉴定。PCR产物进行T7 endonuclease I酶切验证结果如图1B，其中EGFR C797+6C组的T7 endonuclease I酶切割效率最高（图1C）。由此确定最佳的sgRNA靶向序列为GTTCCCGGACATAGTCC。

**图1:**
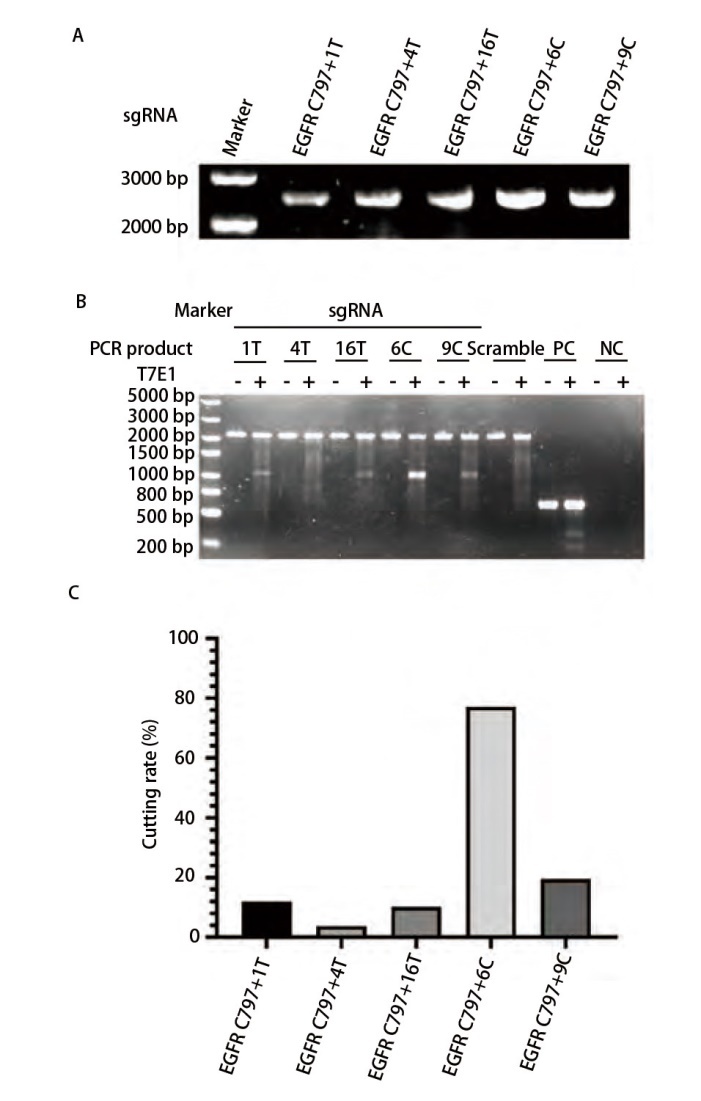
sgRNA的构建及筛选结果。A：各sgRNA质粒回收纯化后琼脂糖凝胶电泳验证结果；B：各sgRNA在HEK-293T细胞上编辑效率验证结果；C：各sgRNA在HEK-293T细胞上编辑效率验证灰度扫描结果。

### 2.2 PC-9 EGFR^D19/T790M/C797S^突变工程细胞株的构建与鉴定

根据上述体系将sgRNA和携带敲入模板的ssDNA共转染PC-9细胞，经Osimertinib筛选和单克隆接种，最终共挑选出16株单克隆细胞株。利用MTS方法评价各单克隆细胞株以及PC-9对EGFR^D19/T790M/C797S^的抑制活性弱的第三代EGFR-TKIs Osimertinib和对EGFR^D19/T790M/C797S^抑制活性强的在研第四代EGFR-TKIs BI4020的敏感性差异，Osimertinib及BI4020对各单克隆的增殖抑制活性测试结果如表2。利用CRISPR/Cas9技术敲入HDR模板序列后，各单克隆对Osimertinib的敏感性相较于PC-9均下降，表明所有单克隆细胞株均可能具有EGFR^D19/T790M/C97S^突变，而Clone 1、2、3、5、6、8、9、11、16对BI4020的敏感性相较于PC-9有所下降，分析可能由于是CRISPR/Cas9的脱靶效应。将Osimertinib敏感性相较于PC-9下降100倍以上，即增殖抑制IC_50_>1000 nmol/L的单克隆细胞株认为是较为合适的单克隆，Osimertinib在16个单克隆中有9个的增殖抑制IC_50_>1000 nmol/L，其中Clone 4 BI4020的IC_50_值最低[（4.88±0.14）nmol/L]，故确认PC-9 EGFR^D19/T790M/C797S^ Clone 4为最适于评价EGFR-TKIs的单克隆，命名为PC-9 EGFR^D19/T790M/C797S^。基因组DNA测序及ICE分析结果（图2）显示该单克隆具有EGFR^T790M/C797S^突变，其CRISPR编辑效率为79%，HDR模板的敲入效率为17%，非同源末端修复的比例为62%且均为移码突变，其中发生缺失突变的比例为51%，+1位点插入突变比例为11%。

**表2:** PC-9及PC-9 EGFR^D19/T790M/C797S^各单克隆对Osimertinib和BI4020的敏感性测试结果（Mean±SD, n=3）

Cell	IC_50_ (nmol/L)	
	Osimertinib	BI4020
PC-9	9.66±0.33	42.27±0.94
PC-9 EGFR^D19/T790M/C797S^ Clone 1	2180.67±141.00	80.29±22.71
PC-9 EGFR^D19/T790M/C797S^ Clone 2	2197.33±255.00	150.40±24.23
PC-9 EGFR^D19/T790M/C797S^ Clone 3	2454.67±384.94	111.84±21.47
PC-9 EGFR^D19/T790M/C797S^ Clone 4	3237.33±169.22	4.88±0.14
PC-9 EGFR^D19/T790M/C797S^ Clone 5	2474.33±80.03	58.00±3.78
PC-9 EGFR^D19/T790M/C797S^ Clone 6	2079.00±240.76	61.25±6.56
PC-9 EGFR^D19/T790M/C797S^ Clone 7	1971.00±164.72	39.76±6.12
PC-9 EGFR^D19/T790M/C797S^ Clone 8	2460.50±152.03	101.31±19.37
PC-9 EGFR^D19/T790M/C797S^ Clone 9	429.53±53.13	58.62±11.56
PC-9 EGFR^D19/T790M/C797S^ Clone 10	252.10±10.62	30.81±6.97
PC-9 EGFR^D19/T790M/C797S^ Clone 11	1195.57±423.73	279.87±21.79
PC-9 EGFR^D19/T790M/C797S^ Clone 12	221.87±44.92	26.37±1.29
PC-9 EGFR^D19/T790M/C797S^ Clone 13	143.00±42.89	20.17±1.30
PC-9 EGFR^D19/T790M/C797S^ Clone 14	361.13±63.62	40.86±1.64
PC-9 EGFR^D19/T790M/C797S^ Clone 15	108.72±25.14	10.18±1.63
PC-9 EGFR^D19/T790M/C797S^ Clone 16	402.67±53.82	44.74±6.57

IC_50_: half maximal inhibitory concentration.

**图2:**
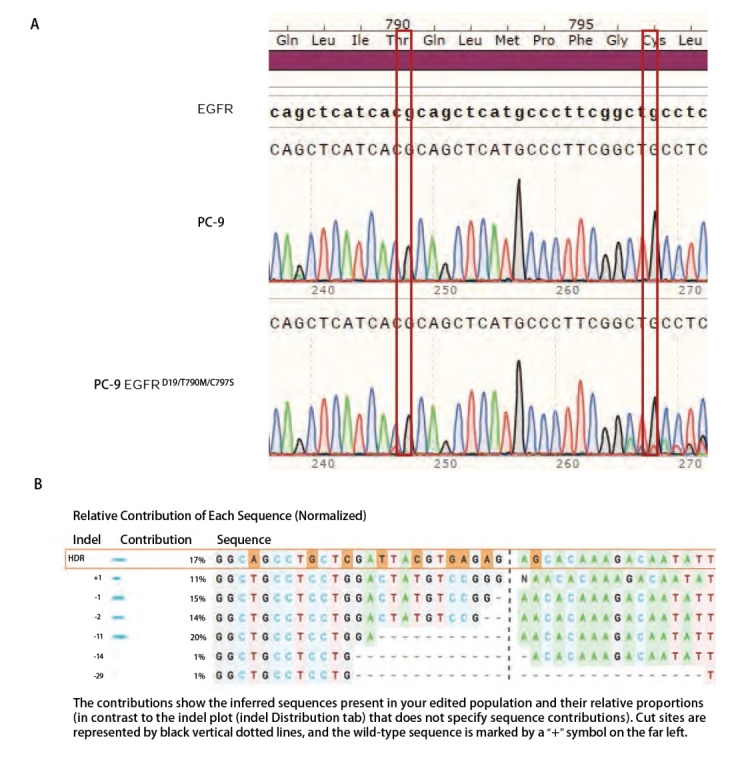
PC-9、PC-9 EGFR^D19/T790M/C797S^基因组DNA PCR产物测序及分析结果。A：PC-9及PC-9 EGFR^D19/T790M/C797S^基因组DNA PCR产物测序结果；B：PC-9及PC-9 EGFR^D19/T790M/C797S^基因组DNA PCR产物序列ICE分析结果。

### 2.3 PC-9 EGFR^D19/T790M/C797S^突变工程细胞株对EGFR抑制剂的响应

#### 2.3.1 EGFR-TKIs对PC-9及PC-9EGFR^D19/T790M/C797S^细胞的增殖抑制作用

利用MTS方法开展细胞增殖能力评价，通过评价化合物对PC-9及PC-9 EGFR^D19/T790M/C797S^细胞增殖抑制能力差异，评估PC-9 EGFR^D19/T790M/C797S^相较于PC-9对不同EGFR-TKIs的敏感性差异。由结果（表3，图3）可以看出，PC-9细胞对针对于EGFR^D19^突变类型有效的EGFR-TKIs均敏感，其中对Afatinib最为敏感，IC_50_为（0.66±0.32）nmol/L，PC-9 EGFR^D19/T790M/C797S^细胞对于Gefitinib、Afatinib、Osimertinib等对EGFR^D19/T790M/C797S^突变类型活性弱的EGFR-TKIs的增殖抑制作用不敏感，IC_50_值相较于PC-9分别增长到了325.20、4656.81和335.10倍；而对于TQB3804、BI4020等对EGFR^D19/T790M/C797S^具有较好抑制活性的EGFR-TKIs的增殖抑制作用敏感，其中对BI4020最为敏感，IC_50_为（4.88±0.14）nmol/L。该结果提示PC-9 EGFR^D19/T790M/C797S^中EGFR^D19/T790M/C797S^对该细胞增殖的影响占主导作用，PC-9 EGFR^D19/T790M/C797S^中最主要的EGFR突变类型为EGFR^D19/T790M/C797S^突变。

**表3:** EGFR-TKIs对PC-9及PC-9 EGFR^D19/T790M/C797S^增殖抑制（Mean±SD, n=3）

Compounds	Generation	IC_50 _(nmol/L)		Ratio （to PC-9）
		PC-9	PC-9 EGFR^D19/T790M/C797S^	
Gefitinib	I	30.75±3.41	>10,000.00	>325.20
Afatinib	II	0.66±0.32	3087.00±65.83	4656.81
Osimertinib	III	9.66±0.33	3237.33±169.22	335.10
TQB3804	IV	151.17±5.30	116.13±8.43	0.77
BI4020	IV	42.27±0.94	4.88±0.14	0.12

TKIs: tyrosine kinase inhibitors.

**图3:**
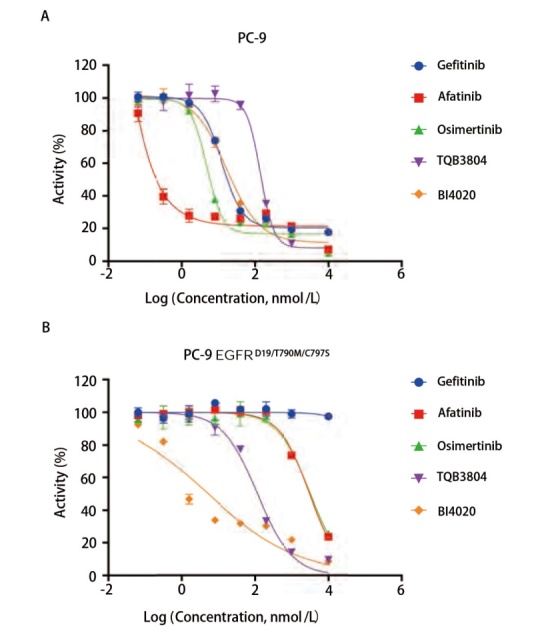
EGFR-TKIs对PC-9及PC-9 EGFR^D19/T790M/C797S^细胞活力的影响。A：EGFR-TKIs作用72 h对PC-9细胞活力的影响；B：EGFR-TKIs作用72 h对PC-9 EGFR^D19/T790M/C797S^细胞活力的影响。

#### 2.3.2 EGFR-TKIs对PC-9及PC-9EGFR^D19/T790M/C797S^细胞的EGFR/AKT/MAPK磷酸化抑制作用

EGFR通过磷酸化激活下游AKT和MAPK信号通路，刺激AKT和MAPK磷酸化从而促进细胞增殖，利用Western blot方法评价化合物对PC-9及PC-9 EGFR^D19/T790M/C797S^细胞的EGFR/AKT/MAPK磷酸化抑制能力，结果（图4）表明对EGFR^D19^突变类型有效的第一代EGFR-TKIs Gefitinib、第二代EGFR-TKIs Afatinib、第三代EGFR-TKIs Osimertinib、在研第四代EGFR-TKIs TQB3804和BI4020在40与200 nmol/L的浓度下均能够显著抑制EGFR/AKT/MAPK的磷酸化。而在PC-9 EGFR^D19/T790M/C797S^细胞上，仅对EGFR^D19/T790M/C797S^突变类型有效的TQB3804和BI4020在40与200 nmol/L的浓度下均能够显著抑制EGFR/AKT/MAPK的磷酸化，该结果表明PC-9 EGFR^D19/T790M/C797S^中EGFR突变类型主要为EGFR^D19/T790M/C797S^，且对该突变类型有效的EGFR-TKIs可抑制该细胞中EGFR及下游AKT/MAPK的磷酸化。

**图4:**
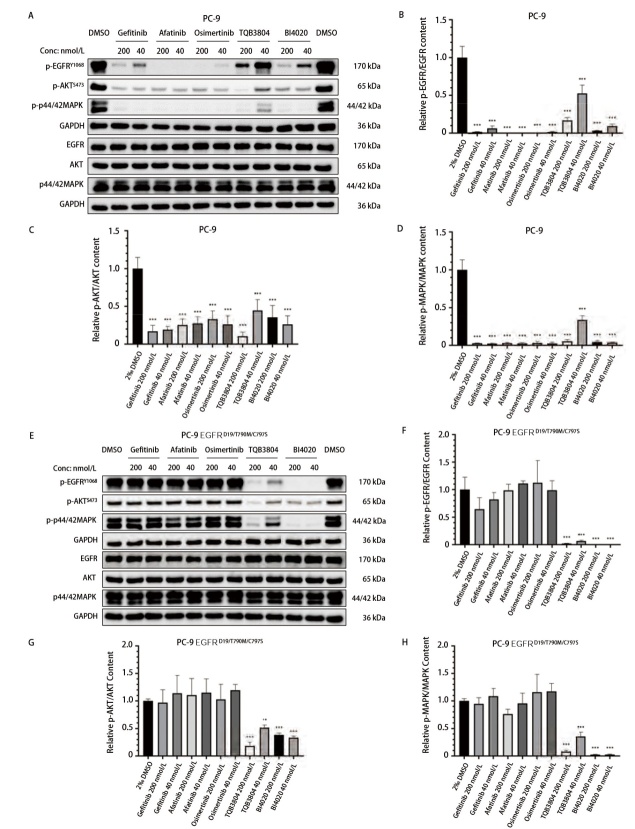
EGFR-TKIs对PC-9及PC-9 EGFR^D19/T790M/C797S^中EGFR/AKT/MAPK磷酸化的影响。A：EGFR-TKIs对PC-9细胞EGFR/AKT/MAPK磷酸化抑制作用；B：EGFR-TKIs对PC-9细胞中EGFR磷酸化抑制作用灰度扫描结果；C：EGFR-TKIs对PC-9细胞中AKT磷酸化抑制作用灰度扫描结果；D：EGFR-TKIs对PC-9细胞中MAPK磷酸化抑制作用灰度扫描结果；E：EGFR-TKIs对PC-9 EGFR^D19/T790M/C797S^细胞中EGFR/AKT/MAPK磷酸化抑制作用；F：EGFR-TKIs对PC-9 EGFR^D19/T790M/C797S^细胞中EGFR磷酸化抑制作用灰度扫描结果；G：EGFR-TKIs对PC-9 EGFR^D19/T790M/C797S^细胞中AKT磷酸化抑制作用灰度扫描结果；H：EGFR-TKIs对PC-9 EGFR^D19/T790M/C797S^细胞中MAPK磷酸化抑制作用灰度扫描结果。

## 3 讨论

本研究利用CRISPR/Cas9技术成功构建了贴近临床的具有EGFR^D19/T790M/C797S^突变类型的PC-9 EGFR^D19/T790M/C797S^细胞，并可用于新一代靶向EGFR耐药突变的创新药物的研发。CRISPR/Cas9是由RNA指导的Cas9核酸酶对靶向基因进行编辑的技术，考虑到不同序列的sgRNA的编辑效率会不同，因此本研究设计并构建了6个序列不同的sgRNA，并且采用HEK-293T工具细胞进行了编辑效率的验证，从而筛选出编辑效率最高的sgRNA EGFR C797+6C用于后续细胞模型的构建。细胞修复切割产生的DNA断裂时，可以引入突变，从而实现基因的敲除、插入或替换，因此将携带HDR模板序列的单链DNA（single-tranded DNA, ssDNA）与sgRNA和Cas9质粒同时转入细胞，可同时实现基因的替换。脱靶基因组编辑是一种在使用工程核酸酶时发生的意外和非特异性基因修饰，脱靶基因组编辑会混淆并削弱CRISPR/Cas9的治疗潜力，这种效应最终会导致有害事件，例如不需要的DNA损伤、免疫反应和细胞毒性^[22]^。脱靶效应通常是sgRNA的非特异性导致，因此本研究在设计sgRNA后利用脱靶效率预测工具进行了分析，筛选出脱靶效率较低的sgRNA序列。为避免替换后的基因片段被sgRNA重复识别，本研究设计HDR模板序列中除EGFR^T790M/C797S^突变类型的序列外，还在不改变编码蛋白序列的前提下将sgRNA识别的关键位点的基因序列进行了编辑，并在两端保留同源序列。EGFR中T790M/C797S突变分为顺式和反式构象，目前未有针对T790M/C797S顺式构象的批准药物，因此本研究计划所构建的细胞株中EGFR^T790M/C797S^突变为顺式构象，但不足之处是本研究未能对其实际的构象进行验证，其可能同时具有顺式和反式两种构象。本研究采取ssDNA的HDR模板，相较于双链DNA（double-stranded DNA, dsDNA）的HDR模板，除操作简单外还可提高编辑效率，降低细胞毒性。PC-9中具有多个EGFR拷贝，而CRISPR/Cas9技术的编辑效率通常无法达到100%，结合测序结果，本研究未能得到一个纯合的表达EGFR^D19/T790M/C797S^单一突变类型的细胞模型，这是CRISPR/Cas9本身编辑效率导致的。根据测序结果使用ICE平台分析预测，所得细胞株中，17%的EGFR等位基因成功敲入了T790M和C797S突变，21%的等位基因保持不变，62%的等位基因因移码突变而失活，这也导致最终挑选的PC-9 EGFR^D19/T790M/C797S^细胞株中EGFR表达水平较亲本发生显著下调。该细胞株同时携带EGFR^D19^敏感突变和EGFR^D19/T790M/C797S^耐药突变，这与临床中的结果更为接近。

目前市场上已有3代EGFR-TKIs上市，而不同EGFR-TKIs在敲入突变的携带EGFR^D19/T790M/C797S^突变的细胞中的表现的研究仍较少。通过检测经典第一代至第三代EGFR抑制剂和在研第四代抑制剂在该细胞模型以及亲本PC-9细胞上对EGFR激活及下游AKT/MAPK信号通路的影响，发现在PC-9细胞上，对EGFR^D19^突变类型有效的第一代EGFR抑制剂Gefitinib，第二代EGFR抑制剂Afatinib，第三代EGFR抑制剂Osimertinib以及在研第四代EGFR抑制剂TQB3804和BI4020均能抑制细胞的增殖且显著抑制EGFR及下游AKT/MAPK的磷酸化。在所构建的PC-9 EGFR^D19/T790M/C797S^细胞株上，对EGFR^D19/T790M/C797S^耐药突变活性较弱的Gefitinib、Afatinib及Osimertinib对该细胞的增殖抑制作用及EGFR信号通路的抑制作用均较弱，而仅有在研第四代EGFR-TKIs可显著抑制该细胞的增殖和EGFR信号通路的激活。以上结果表明，本研究所构建的PC-9 EGFR^D19/T790M/C797S^细胞株依赖EGFR^D19/T790M/C797S^的激活进行细胞增殖，对不同类型的EGFR抑制剂表现出不同的敏感性，并且依托该细胞所搭建的活性评价和机制验证体系可用于新一代靶向EGFR耐药突变的创新药物的研发。另外，本研究还有不足之处，首先虽然本研究在设计sgRNA序列以及HDR模板时，采取了一系列措施尽可能降低了脱靶效应的产生，但实际运用中，CRISPR/Cas9的脱靶效应是难以完全避免的，因此本研究所构建的细胞株需在后续研究中进行全基因组测序以进行脱靶分析。其次，本研究侧重于在体外水平为新一代靶向EGFR突变耐药的创新药物研发提供了细胞水平的活性评价和验证体系，在后续研究中将会建立PC-9及所构建细胞株的荷瘤裸鼠模型，评估各代EGFR-TKIs对荷瘤裸鼠瘤重、瘤体积的影响，以及检测肿瘤组织中EGFR以及下游信号通路的磷酸化水平抑制情况，完成体内水平的验证工作，从而为新一代靶向EGFR耐药突变的创新药物研发提供体内水平的活性评价和验证体系。

现有上市EGFR-TKIs如Gefitinib、Afatinib及Osimertinib对携带EGFR^D19/T790M/C797S^的肿瘤细胞不敏感，需要开发更有效的新型EGFR抑制剂进行治疗。由于EGFR^D19/T790M/C797S^突变是Osimertinib的主要耐药机制之一，针对该突变所开发的第四代EGFR-TKIs大多正处于临床或临床前阶段，目前仍未有批准用于该突变类型患者的一线治疗药物。因此，本研究所构建的贴近临床的具有EGFR耐药突变的NSCLC细胞模型对进一步探讨EGFR-TKIs的应用及新一代靶向EGFR耐药突变的创新药物的研发具有重大意义。
